# c-Met and Other Cell Surface Molecules: Interaction, Activation and Functional Consequences

**DOI:** 10.3390/biomedicines3010046

**Published:** 2015-01-15

**Authors:** Giuditta Viticchiè, Patricia A. J. Muller

**Affiliations:** MRC (Medical Research Council) Toxicology Unit, Lancaster Road, Leicester LE1 9HN, UK; E-Mail: gv34@le.ac.uk

**Keywords:** c-Met, HGF receptor, RTK, recycling, crosstalk

## Abstract

The c-Met receptor, also known as the HGF receptor, is one of the most studied tyrosine kinase receptors, yet its biological functions and activation mechanisms are still not fully understood. c-Met has been implicated in embryonic development and organogenesis, in tissue remodelling homeostasis and repair and in cancer metastasis. These functions are indicative of the many cellular processes in which the receptor plays a role, including cell motility, scattering, survival and proliferation. In the context of malignancy, sustained activation of c-Met leads to a signalling cascade involving a multitude of kinases that initiate an invasive and metastatic program. Many proteins can affect the activation of c-Met, including a variety of other cell surface and membrane-spanning molecules or receptors. Some cell surface molecules share structural homology with the c-Met extracellular domain and can activate c-Met via clustering through this domain (e.g., plexins), whereas other receptor tyrosine kinases can enhance c-Met activation and signalling through intracellular signalling cascades (e.g., EGFR). In this review, we provide an overview of c-Met interactions and crosstalk with partner molecules and the functional consequences of these interactions on c-Met activation and downstream signalling, c-Met intracellular localization/recycling and c-Met degradation.

## 1. Introduction

c-Met, also known as the scatter factor receptor, is one of the major players in invasive growth [1] and morphogenetic events during embryogenesis and adult life [2]. c-Met was first identified as a member of the receptor tyrosine kinases family (RTKs) in 1987 [3] and has since attracted widespread interest because of its aberrant activation during the malignant progression of various cancers. Upon binding to its ligand, hepatocyte growth factor (HGF), c-Met triggers the induction of several downstream signalling cascades leading to a multitude of outcomes: increased survival, proliferation, anchorage-independent growth, enhanced motility, migration, scattering, metastasis and invasion [4,5,6,7,8,9,10].

## 2. c-Met Structure and Activation

**Figure 1:**
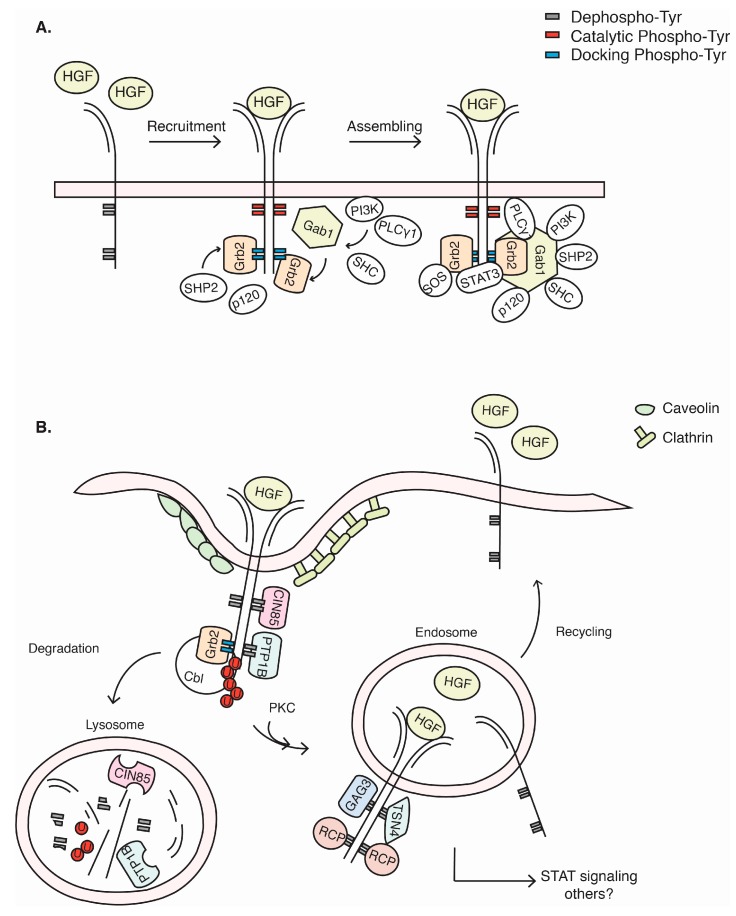
Activation, signalling, internalization and recycling of c-Met. (**A**) Representation of the activation of the c-Met heterodimer illustrating the catalytic and docking tyrosine residues in the non-phosphorylated inactive state, non-HGF-bound state (**left**) or the active state (**right**). HGF-mediated c-Met activation triggers the sequential trans-phosphorylation of catalytic tyrosines (Tyr1234-1235, red residues) and docking tyrosines (Tyr1349-1356, blue residues), determining the biochemical signature for the further recruitment of amplifier and transducer molecules. Grb2 and STAT3 directly associate with the c-Met carboxy-terminal tail, while Gab1 interacts both indirectly (through Grb2) or directly providing a docking structure for SHC, PI3K, SHP2, PLCγ1 and p120, resulting in activation of the downstream signalling pathways; (**B**) Representation of the internalization and recycling of c-Met. To prevent c-Met over-stimulation, several protein-tyrosine phosphatases downregulate the c-Met signal, reverting the catalytic and docking tyrosines to a non-phosphorylated inactive state. The dissociation of the intermediate molecules from the complex refines the tuning and timing of the c-Met-mediated biological response and allows further internalization of c-Met. The Cbl E3 ubiquitin ligase mediates ubiquitination of c-Met, providing a signal for c-Met internalization, which has been shown to be clathrin- or caveolin-dependent. Internalization can be enhanced by Grb2, CIN85, SNX2, CD44v6 and PTP1B. Once internalized, the c-Met receptor can be delivered to lysosomes to be degraded or it can be recycled back to the plasma membrane through the endosomal compartments. PKCε is important for the delivery towards the endosomes, from which the c-Met receptor can signal to specific signalling routes, including STAT3. Recycling back to the plasma membrane has been shown to be dependent on RCP, GGA3 and/or TSN4.

c-Met is a heterodimer consisting of an extracellular α-chain bound through a disulphide bridge to a transmembrane β-chain [11]. The structure comprises several domains, including a SEMA domain (semaphorin like domain), a PSI domain (plexin-semaphorin-integrin domain) and four IPT domains (immunoglobulin–plexin–transcription factor domain) that share structural homologies with plexins, integrins, semaphorins and immunoglobulins [12]. As soon as HGF is recognized by the immunoglobulin-like domains, two c-Met heterodimers dimerize, leading to the autophosphorylation of two tyrosine residues within the catalytic loop (Tyr1234–Tyr1235). Subsequently, further autophosphorylation of two tyrosines (Tyr1349–Tyr1356) in the *C*-terminal domain provide the docking platform for the recruitment of other molecular interactors and signal conveyors [13] (Figure 1). Among these are the Grb2-associated binding protein 1 (Gab1) that supplies binding sites for Src–homology-2 domain (SH2)-containing effectors, like the SH2-transforming protein (SHC), “the phosphoinositide 3 kinase (PI3K), the SH2-domain containing protein tyrosine phosphatase (SHP2), the phospholipase Cγ1 (PLCγ1), the signal transducer and activator of transcription 3 (STAT3) and the Ras GTPase p120 [7,8,9,14,15,16]. Most of the cellular responses induced by c-Met activation are mediated by the action of the adaptor molecule, Gab1 [9,17,18]. A sequence of 13 amino acids, identified as the c-Met binding site, is responsible for the direct association of Gab1 to c-Met, leading to the assembly of the docking platform [7,17,18]. Gab1 activity and function are enhanced and prolonged upon HGF-dependent phosphorylation of tyrosine residues in Gab1 that provide extra binding sites for the recruitment of the aforementioned signal transducer and docking proteins. Consequently, by acting as a molecular bridge to unleash c-Met signalling, Gab1 functions as an upstream initiator of the Ras–ERKs/MAPKs cascade.

To regulate the intensity of the c-Met signal, another group of proteins regulate the duration of c-Met activity through dephosphorylation of the catalytic and docking tyrosines. These phosphatase tyrosine proteins (PTPs), including the receptor, PTPs density-enhanced phosphatase 1 (DEP1), the non-receptor, PTPs PTP1B, leukocyte common antigen-related (LAR) and T-cell PTP (TCPTP), act as antagonistic c-Met partners in order to prevent prolonged c-Met signalling [19,20,21].

## 3. c-Met Signalling Cascade

Activation and crosstalk between several signalling pathways has been described following stimulation of the c-Met receptor with its ligand, HGF, leading to a global change in gene expression [22,23].

The mitogen-activated protein kinases (MAPKs) and the extracellular signal-regulated kinases (ERK1/2) are among the major c-Met executors downstream of c-Met activation and interaction with docking molecules [24]. Two main transducer pathways have been shown to link phosphorylated c-Met to the MAPK/ERK components; one involving activation of Ras small GTPase following the association of Grb2-son of sevenless protein (SOS) complex to the c-Met *C*-terminus [13] and one involving Ras inhibiting protein p120 deactivation upon c-Met–Gab1–SHP2 interaction [25]. Both of these steps result in translocation of ERKs to the nucleus to promote ETS/AP1-mediated transcriptional regulation of cell cycle modulators and adhesion proteins in order to control cell proliferation and motility [5,6,7,8,9,26]. c-Met can also activate the Jun amino-terminal kinases (JNKs) and p38 MAPKs via the same pathways, thereby regulating cytoskeleton-associated proteins that are important in cell migration and scattering [10,27]. Alternatively, PI3K was found to be directly activated by c-Met or indirectly by Ras-driven Akt/PKB signalling, which, in turn, leads to BCL2 antagonist of cell death (Bad) inactivation and to MDM2-mediated p53 degradation, promoting enhanced cell survival [28,29,30]. STAT3 associates with phosphorylated c-Met and undergoes phosphorylation itself, after which it translocates into the nucleus and increases transcription of genes involved in tumourigenesis [31,32].

## 4. c-Met Endocytosis and Recycling

As with all growth factor-induced receptors, ligand-induced signalling through c-Met is tightly controlled. One of the control mechanisms comprises the rapid internalization and degradation/recycling of the receptor in a process called receptor-mediated endocytosis [33]. The rapid removal of the receptor from the plasma membrane was described as an important mechanism by which cells quickly switch off further signalling to prevent sustained stimulation of the receptor, which can otherwise promote transformation [33]. Ligand stimulation of c-Met leads to polyubiquitinated c-Met and proteasomal degradation upon c-Met internalization [34]. Petrelli *et al.* demonstrated that internalization and degradation was dependent on c-Met binding to the endophilins, CIN85 and Cbl, through a process of clathrin-coated vesicle formation [35]. However, more recently, clathrin-independent endocytosis of the c-Met receptor through interaction with caveolin has also been demonstrated [36]. A pivotal role for Cbl in c-Met degradation was confirmed by Li *et al.* and provided evidence that the signalling adaptor protein, Grb2, is required for c-Met endocytosis [37]. Other factors involved in c-Met internalization are dorsal ruffle formation [38], sorting nexin 2 (SNX2) [39] and the c-Met interaction partner, CD44v6 [40] (see the CD44 section). Upon internalization, the endoplasmic reticulum-localized protein-tyrosine phosphatase 1B (PTP1B) and dynamin were found to coordinate the early events of endosome formation that dictate c-Met trafficking and degradation [34,41,42]. The fact that Cbl can promote degradation, but is not required for internalization [37], suggests that c-Met endocytosis and c-Met degradation are different processes that might serve different purposes. Indeed, it has been suggested that the compartmentalization of growth factor receptors plays a crucial role in the signal transduction process and that rather than being just destined for irreversible degradation, the endocytic vesicles might provide a modulatory substation that retains a targeted receptor to act at the right time at the right place with the right signalling output [43]. Evidence that c-Met localization determines signalling is provided by Kermogant *et al*., who showed that the translocation of c-Met from the endosomal compartment to the perinuclear compartment is dependent on PKCα, and inhibition of PKCα resulted in a decreased signalling of c-Met to STAT3 [44]. The same group also revealed that c-Met signalling to Rac1 is differently regulated from c-Met localized in peripheral endosomes than in perinuclear endosomes [45]. Trafficking to the perinuclear regions depends on an interaction with PKCε, which was demonstrated to be important for *p*-ERK1/2 accumulation at focal complexes upon c-Met activation [46].

Furthermore, various groups have provided evidence that endosomal-localized c-Met is not just destined to be degraded, but can be returned to the plasma membrane in a process that is called receptor recycling. Receptor recycling has long been demonstrated for another RTK, EGFR [47], and provides a means to sustain downstream signalling. Using c-Met mutant proteins that were found in renal cancers, Joffre *et al.* showed that the mutant versions of c-Met underwent an increased Cbl–Grb2-dependent recycling and a concomitant aberrant activation of GTPase Rac1, leading to enhanced cell migration, anchorage-independent cell growth and *in vivo* tumorigenesis [48]. Proteins that have been implicated in recycling back to the plasma membrane include Hrs, Golgi-localized γ-ear-containing Arf-binding protein 3 (GGA3), Tensin-4 and Rab coupling protein (RCP) [49,50,51,52] (Figure 1). The proteasomal inhibitor, lactacystin, promotes c-Met recycling back to the plasma membrane, possibly through preventing c-Met from entering the lysosomal degradation pathway. Lactacystin treatment coincided with a decrease in the endosomal sorting protein, Hrs, and siRNA-mediated Hrs inhibition promoted c-Met activity, suggesting that Hrs dictates c-Met degradation [49]. The adaptor protein, GGA3, interacts with activated c-Met to promote its recycling, and loss of GGA3 resulted in attenuated ERK1/2 activation and pronounced c-Met degradation [52]. Tensins are scaffold proteins that are known for their role in coupling integrin receptors to the cytoskeleton. TNS4, unlike the other tensins, abrogates this link and promotes cell migration. TNS4 was found to also interact with c-Met and to promote its recycling to the plasma membrane to prevent degradation in an integrin-independent manner [50]. Remarkably, we identified that another integrin binding partner, RCP, could also promote c-Met recycling in cells dependent on the expression of an oncogenic mutant p53 protein [53]. Loss of RCP in mutant p53-expressing cells decreased the recycling of c-Met back to the plasma membrane, thereby attenuating ERK1/2 signalling and decreasing cell invasion and cell scattering. These data suggest that the imbalance between recycling and degradation in favour of continuous endosomal trafficking contributes to the maintenance of the activated state of c-Met, leading to pro-malignant signalling.

## 5. c-Met and Its Membrane-Spanning Partner Molecules

**Figure 2:**
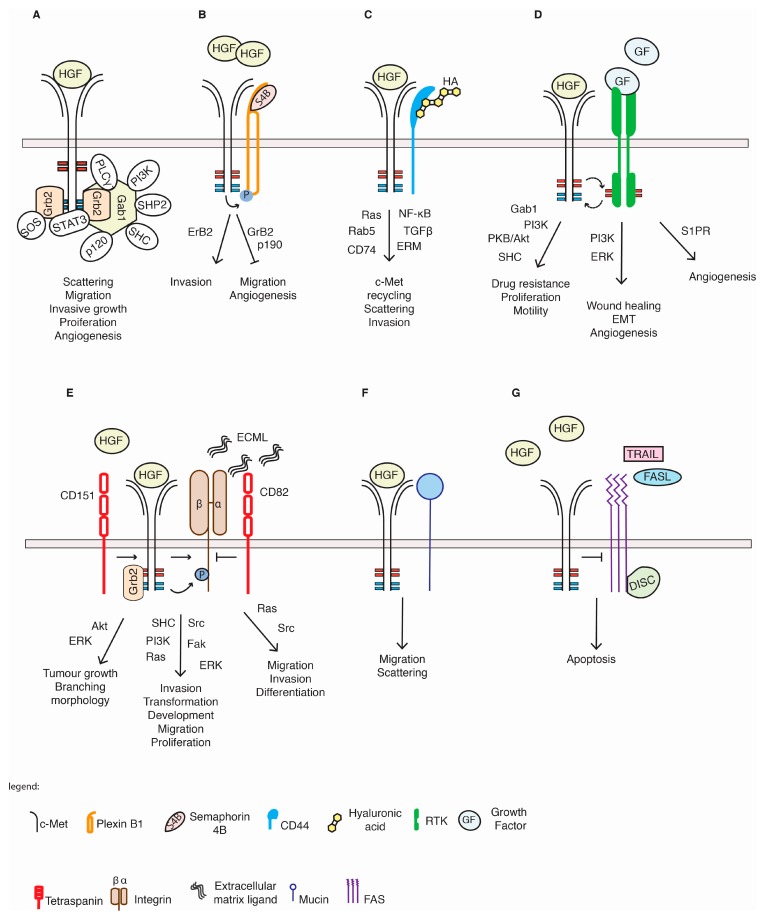
Membrane molecules that interact with and influence c-Met function. c-Met interacts with a variety of other cell membrane molecules and receptors that can modulate c-Met signalling and cellular outcome. (**A**) Signalling of the c-Met dimer alone; (**B**) The plexin B1 extracellular domain has been found to associate with the c-Met counterpart, enhancing ErB2-mediated invasion or repressing c-Met through GrB2-p190 to promote invasion, migration and/or angiogenesis; (**C**) CD44 family members physically bind to c-Met to promote its internalization, which promoted c-Met-dependent invasion and migration; (**D**) Among the RTKs, the EGFR association with c-Met has been most studied. Many RTKs can transphosphorylate c-Met tyrosine residues and thereby amplify c-Met-signalling to promote drug resistance, EMT or wound healing. EGFR also promotes c-Met ectodomain shedding, which is not depicted in this figure; (**E**) Integrins associate with c-Met, enhancing its transforming potential. In detail, HGF and extracellular matrix proteins participate in c-Met/integrin binding, promoting migration and proliferation in a Ras- or Src-dependent manner. Tetraspanins were found to modulate integrin/c-Met function; (**F**) Mucins inhibit c-Met via unknown mechanisms, leading to decreased HGF-driven migration and scattering; (**G**) Inactive c-Met can prevent FasL- or TRAIL-driven Fas or DR5 complex formation, with TRAIL resulting in decreased apoptotic signalling.

c-Met signalling, degradation, activation and intracellular localization are not only determined by the docking and signalling molecules described so far, but can also be modulated by a large variety of c-Met interacting molecules comprising membrane spanning proteins and receptors, which include plexins, integrins, semaphorins and other RTKs (Table 1 and Figure 2). These proteins interact with c-Met and potentiate, inhibit or modulate the downstream signalling of c-Met, as explained below.

**Table 1:** c-Met interacting proteins and their function on c-Met.

Receptor		Cell System	Effect on c-Met	Biological Response	Reference
Plexins	Plexin B1	HUVEC	Inhibition	↓ Angiogenesis	[54]
		HT-29	Activation	↑ Invasion	[55]
		SK-BR3, MLP29	Activation	↑ Migration ↑ Colony formation, ↑ Invasive growth	[56]
		YUSIK, MDA-MB 468, MCF-7	Inhibition	↓ Migration	[57,58,59,60]
	Plexin B3	HUVECs	Activation	↑ Migration	[61]
CD44	CD44v9	C4-2, LNCap	Activation	↑ Resistance, invasion	[62]
	CD44v6	WM9, WM164, 1205Lu	Activation	↑ Migration	[63]
		HeLa, HT29, HepG2	Activation	↑ c-Met internalization, signalling, scattering	[40,64,65]
		fibroblasts	Activation	↑ Proliferation	[66]
	CD44v10	Human pulmonary microvascular EC, B-cells	Activation	↑ EC barrier enhancement, B-cell survival	[67,68]
Tetraspanin	CD151	AccM, Acc2	Activation	↑ Migration, proliferation	[69]
		MDA-MB-231	Activation	↑ Branching morphogenesis	[70]
		GTL-16	Activation	↑ Proliferation, anchorage-independent growth	[71]
	CD82	PC3, Hepa1-6	Inhibition	↓ Migration, invasion	[72,73]
		Oligodendrocytes (O4^+^ cells)	Inhibition	↓ Differentiation	[74]
		HCV29/YTS1	Inhibition	↓ Invasion	[75]
		H1299	Inhibition	↓ Migration, lamellipodia formation	[76]
Integrin	α6β4	GTL-16, A431, MDA-MB-435	Activation	↑ Invasive growth	[71,77]
		MEFs	Activation	↑ Colony formation, tumour growth	[78]
		DU145	Activation	↑ Self-renewal, invasion	[79]
		HLMVEC, HPAEC	Activation	EC barrier integrity	[80]
	α5β1	HMVEC	Activation	↑ Migration, proliferation	[81]
		SKOV3ip1, HeyA8	Activation	↑ Metastasis	[82]
	α3β1	Mouse papillary cells	Activation	Kidney morphogenesis	[83]
	αxβ1	PC9	Activation	↑ Proliferation	[84]
	α2β1	PMCs	Activation	↑ PMC activation	[85]
RTKs	Ron	NIH3T3	Reciprocal Activation	↑ Colony formation	[86]
	EGFR	A431, HepG2, AKN-1, HuH6, MRC5	Activation	↑ c-Met signalling	[87]
		PyVmT, MDA-MB231, 4T1, NCl H596, DLD1, HT29	Activation	↑ Motility, proliferation	[88,89,90]
		PC-9, HCC827, SNU-16, MKN45, BT474, SKBR3	Activation	↑ Drug resistance	[91,92,93]
		5637 tumour bladder cell line	Activation	↑ Survival, cell growth	[94]
		ARPE-19	Activation, Ecto-domain shedding	↑ Wound healing	[95]
		A549	Ecto-domain shedding	NA	[96]
		H1993, EBC1	Activation	↑ Survival, proliferation	[97]
		H1975, H520, A549	Activation	↑ Tumour growth and survival	[98]
		GEO-CR, SW48-CR	Activation	↑ c-Met phosphorylation, ↑ Survival	[99]
		32D, PC9	Activation	↑ c-Met phosphorylation, metastasis, invasion and colony formation	[100]
		201T, A549	Activation	↑ c-Met phosphorylation, xenograft growth	[101]
	Her2	SK-BR3, BT474	Activation	↑ Drug resistance	[102]
		H1993, EBC1	Activation	↑ Survival, proliferation, ↑ Migration	[97]
		MDCK	Activation	↑ EMT	[103]
	Her3	H1993, EBC1	Activation	↑ Survival, proliferation	[97]
		HCC827,	NA (HER3 activation) *	↑ Drug resistance	[104]
		MKN45, GTL16	Activation	↑ Drug resistance	[91,105]
	IGFR	L3.6pl	Activation	↑ Migration, invasion	[106]
	RET	H1993, EBC1	Activation	↑ Migration	[97]
Death receptors	Fas	HepG2, Hepa1-6	No effect	↓ Apoptosis	[107,108]
		HUVECs	NA	↓ Apoptosis	[109]
	DR5	Medulloblastoma/glioma cell lines	NA	↓ Apoptosis	[110]
Mucins	Muc1	Panc-1, HPAF2, MDA-MB-435, Mahlavu, SNU-449	Inhibition	↓ Invasion, EMT	[111,112,113]
	Muc20	HEK293, CHO-K1	Inhibition	↓ Invasion, EMT	[114]
NRP1		PCa cells	Activation	↑ Bone metastasis	[115]
ICAM1		HT29, HepG2	Activation	↑ Proliferation	[116]

* Cooperation between c-Met and EGFR was seen, but c-Met activation was not directly determined.

### 5.1. Plexin Proteins

Plexins are transmembrane receptors originally discovered as main regulators of semaphorin signalling [117]. They activate downstream signalling pathways, related to cytoskeleton remodelling, via a variety of specific small Rho GTPases, determining cell migration and invasion [118,119]. In the context of RTK crosstalk, the high structural homology of the extracellular domain with plexins, which include a propeller structure involved in protein-protein interactions, make plexin family members good molecular partners for a variety of RTKs [55]. With respect to c-Met, plexin B1 and its ligand, semaphorin 4D, have been most studied, and both have been implicated in cancer progression [120]. The precise role of the plexin B1/Sema4D signalling axis in the malignant phenotype is still unknown, and several reports demonstrate functions both inhibiting and promoting malignancy [120]. This diversity is also reflected in the observation that plexins have opposing roles on c-Met function. Conrotto *et al.* and Giordano *et al.* both demonstrated a role for Sema4D to promote c-Met activation and signalling through binding with the plexin B1 receptor in epithelial cells, resulting in increased invasion and migration [55,56]. Others, however, revealed in various systems an inhibitory role for plexin B1 on c-Met function through direct binding, leading to decreased cell migration and decreased angiogenesis [54,57,58,59]. In breast cancer cell lines, plexin B1 was found to be phosphorylated by c-Met, leading to Grb2 (growth factor receptor bound-2) recruitment to the plexin B1/c-Met complex [59]. Grb2 subsequently recruited p190 RhoGAP, resulting in RhoA deactivation and suppression of cell migration. The differential function of Sema4D/plexin in tumour biology is also illustrated by the variable results in studies examining the expression levels of these molecules in the disease progression of human cancers with both low Sem4D and high plexin-B1, leading to poorer survival [55,56,60,61].

The differential role of the plexin B1/Sema4D/c-Met signalling axis in cell migration could be related to the expression of the RTK, ErbB2. While plexin B1 triggered increased cell invasion of LNCaP cells in an ErbB2-dependent manner, PC3 cells highly expressing c-Met displayed decreased motility after plexin B1 activation [121]. Similarly, MDA MB468 cells that express high levels of c-Met were inhibited in cell migration upon Sema4D-induced plexin B1 activation, while migration of MCF7 cells that express high levels of ErbB2 was increased under the same circumstances [60]. Interestingly, the effect on MCF7 could be reversed by knockdown of ErbB2 and overexpression of c-Met. In addition, overexpression of ErbB2 diminished the binding of c-Met to plexin B1, suggesting that in breast cancer cells and in prostate cancer cells, ErbB2 and c-Met are competing for plexin B1 binding, which largely determines the cellular outcome of Sema4D signalling. However, ErbB2 expression levels cannot explain the promoting effect of plexin B1 on c-Met function seen by Giordano *et al.* and Conrotto *et al.* [55,56], and it seems likely that other factors must play a role in determining c-Met function in relation to plexin B1 binding. c-Met was also found to interact with another plexin, plexin B3, leading to c-Met activation, leading to increased migration of HUVEC cells [61]. It will be interesting to see whether ErbB2 or other RTKs can influence this interaction and whether c-Met can be regulated by other members of the plexin family.

### 5.2. CD44 Proteins

CD44 glycoproteins are a family of transmembrane proteins transcribed from one mRNA that undergoes alternative splicing to generate multiple isoforms [122]. Ligands for CD44 are hyaluronic acid and osteopontin, which can promote tumour cell growth and chemotaxis, respectively [122]. A role for CD44 in the HGF signalling path was first described by van der Voort *et al.* [123], showing that CD44 promotes HGF signalling through c-Met. Mice that have been engineered to lose CD44 expression display haploinsufficiency for c-Met, demonstrating a role for CD44 and c-Met collaboration *in vivo* [124]. An interaction between c-Met and CD44 proteins was shown by Orian-Rousseau *et al.* revealing that HGF-dependent scattering and invasion of several cancer cell lines and primary cells require the function of the CD44v6 isoform [64]. This CD44v6 isoform was required for c-Met autophosphorylation and downstream signalling through interaction with ERM (Ezrin, radixin and moesin) proteins that link the actin cytoskeleton to the cellular membrane [64,65,125] and Ezrin phosphorylation indeed is required for HGF-induced cell scattering and morphogenesis [125]. Through this link with the ERM proteins, CD44v6 promotes the internalization of c-Met to a rab5-positive endosomal compartment from which c-Met promoted downstream signalling [40]. Some other studies have since confirmed that overexpression of this particular isoform of CD44 in different tumour cell systems promotes c-Met function and is involved in the crosstalk between c-Met and the NF-κB or the TGF-β1 signalling pathways [63,66]. The role of a co-receptor linking c-Met to ERM proteins is not exclusive to CD44v6, as more recently, ICAM1 was shown to fulfil a similar role in the absence of CD44v6 on c-Met function [116].

Other CD44 isoforms have similarly been shown to promote c-Met function. In prostate cancer cell lines, HGF-dependent activation of c-Met stimulated hyaluronan/CD44v9 signalling, which stabilized the androgen receptor and promoted its function [62]. Singleton *et al.* demonstrated that CD44 proteins are important regulators of c-Met-mediated vascular barrier enhancement. This process involves the association of CD44v10 with HGF-activated c-Met and its further translocation into Caveolin-enriched microdomains (CEMs, also named lipid rafts) on the cell membrane [68]. The CD44v10/c-Met then recruits Tiam1, cortactin and/or dynamin 2 proteins, providing the components for proper CEM scaffolding that preserves vascular endothelial cell barrier integrity. In summary, these data demonstrate that various CD44 isoforms have a positive role in c-Met functioning by promoting c-Met coupling to the ERM proteins, interaction with other signalling pathways and c-Met localization to specific microdomains on the cellular membrane.

### 5.3. Integrins

Integrins are heterodimeric membrane receptors responsible for the adhesive interactions between the cell and its surroundings, including the extracellular matrix and neighbouring cells [126]. They exert this scaffold function through the recruitment of a complex network of proteins that connect the actin cytoskeleton to extracellular components [127]. Besides promoting the assembly of tissues with specific physical and mechanical properties, they are also important molecular transducers mediating extracellular stimuli to intracellular signalling pathways. Integrins are composed of an α and β subunit, giving rise to a large number of different integrin molecules that respond to and interact with specific ligands. The heterogeneity in integrin molecules and their ligands is also reflected in the diverse biological functions of these molecules, including cell cycle regulation, modulation of the cell shape, cell motility, invasion and metastasis.

The crosstalk between c-Met and integrins is complex and involves a reciprocal regulation that can be ligand-dependent or -independent and signalling to similar downstream signalling molecules, leading to enhanced activation of those molecules and a c-Met-dependent transcriptional regulation of integrin expression [128].

The best-studied integrin receptor in relation to c-Met signalling is probably the α6β4 receptor, which binds laminin. Although both receptors can individually promote invasion [129], several reports suggest an association between this integrin and c-Met, which amplifies the signalling of both receptors [71,77,78,79,80]. Binding between c-Met and α6β4 promoted phosphorylation of the β4 integrin unit, enabling a subsequent activation of Shc and PI3K. c-Met activation occurred independent of integrin binding to laminin, as a truncated variant of β4 integrin unable to bind the ligand laminin still retained the ability to trigger invasion of cells in a HGF- and c-Met-dependent manner [62,77]. The β4 integrin/c-Met interaction can also impinge on other c-Met functions, as demonstrated in endothelial cells, in which HGF induced the formation of a β4 integrin/c-Met/sphingosine 1-phosphate receptor 1 (S1PR1) complex that was localized at CEMs and promoted vascular integrity [80] or in prostate tumour progenitor cells that underwent self-renewal [79].

c-Met was found to associate with other integrins, including the β1 integrins, and, thereby, promoted an invasive program or induced kidney development [81,82,83,84,85]. Mitra *et al.* reported that in epithelial cells, fibronectin promoted the association between α5β1 integrin and c-Met, leading to c-Met phosphorylation and increased Src and FAK activation [82]. Interestingly, in agreement with previous studies that reported c-Met activation upon cell adhesion in the absence of ligand, α5β1 integrin-mediated c-Met signalling was independent of the presence of HGF [82,130].

Together, these data demonstrate that a variety of integrins and, by implication, cell adhesion can positively influence c-Met signalling. The interaction between other RTKs and integrins has been studied in more detail and revealed more details on how integrins and the extracellular matrix can regulate RTK function [131]. For example, matrix attachment can exert inhibitory functions on EGFR [132], and EGFR signalling seems critically dependent on EGFR co-recycling with the α5β1 integrin in an RCP-dependent manner [133]. Given that RCP can promote recycling of c-Met [51], it is likely that α5β1 integrin can also promote c-Met recycling. Further research is needed to elucidate the precise functional and mechanistic details underlying the integrin and c-Met interaction and their interplay.

### 5.4. Tetraspanins

c-Met was also found to interact with members of the tetraspanin superfamily of membrane-spanning proteins. These molecules are known for their tight association with integrins and can modulate their function, possibly through compartmentalization of integrins [134]. It is therefore not surprising that some tetraspanin molecules can impinge on the c-Met/integrin interactions and regulate c-Met signalling. Klosek *et al.* first showed in human salivary gland cells that CD151 associates with c-Met and α3 or α6 integrin subunits, enhancing HGF/c-Met signalling to promote cell migration and proliferation [69]. Similarly, they found that the CD151 interaction with c-Met in MDA-MB-231 breast cancer cells triggered Akt activation, resulting in branching networks in Matrigel [70]. In GTL16 cells, Franco *et al.* revealed that CD151 associates with the c-Met receptor to drive β4 integrin phosphorylation and facilitated the coupling between c-Met and Gab1–Grb2 to promote MAPK phosphorylation and tumour growth [71].

Conversely, many publications have demonstrated an inhibitory role for the tetraspanin CD82 in c-Met signalling [72,73,74,75,76]. CD82, possibly in combination with gangliosides [73,75], prevents c-Met phosphorylation upon HGF activation, exposure to the extracellular matrix ligands or EGFR transactivation [72,74,76]. This leads to impaired binding of c-Met to Gab1/Grb2 [76], decreased Ras or Src activation [72,74] and a subsequent decrease in migration, invasion or differentiation. As the tetraspanins consist of a large family of proteins that can interact with different integrins, it is likely that other tetraspanin proteins will contribute to c-Met signalling. One likely candidate could be the tetraspanin, CD9, which was shown to contribute to cell migration, to facilitate FAK phosphorylation and to interact with EGFR [135,136].

### 5.5. Other RTKs

As signalling from RTKs is often amplified in human cancers, many therapeutics aim to target and inhibit RTKs. Using such inhibitors, it has become obvious that c-Met plays an important role in activating or potentiating the response of other RTKs or *vice versa* and that amplification or activation of c-Met contributes to chemoresistance. RTKs comprise a family of 58 proteins [137] and the closest related family member of c-Met that c-Met has been shown to associate with is RON [86]. Ligand-dependent hetero-dimerization induced specific transphosphorylation of the respective catalytic and docking sites on either RTK, leading to fully activated downstream signalling pathways and increased colony formation of NIH3T3 cells [86].

A large number of publications describe EGFR and c-Met crosstalk in which either RTK can promote the function of the other RTK and/or converge at similar hubs in their signalling pathways. One of the first reports by Jo *et al.* analysing this crosstalk revealed that in epithelial cancer cells, but not in “normal” liver cells, c-Met was constitutively phosphorylated upon TGFα and EGF exposure in the absence of HGF [87]. The authors proposed that in tumour cells, TGFα-mediated EGFR activation induces EGFR/c-Met interaction, detectable as co-immunoprecipitation and resulting in increased c-Met phosphorylation and signalling [87]. A cooperation between c-Met and EGFR interaction is even more apparent from the large number of reports that study drug resistance after inhibition of either RTK. Cells treated with EGFR inhibitors can acquire resistance through c-Met activation or amplification [93,138]. As an example, Troiani *et al.* confirmed the findings of Jo *et al.* and, in addition, revealed that overexpression of TGF-α could confer resistance to EGFR inhibitors through enhancing the EGFR/c-Met interaction and c-Met phosphorylation [99]. EGFR inhibitors can block HGF-mediated proliferation and motility [88,89], and several studies have highlighted the mutual synergism of c-Met and EGFR in promoting drug resistance, leading to the activation of PI3K/Akt and Gab1 [91,92,93,98,139]. The cellular effect of combination therapy can however be context- and cell-dependent, as demonstrated by Zhang *et al.* using xenograft models. Where H1993-derived tumours reacted to combination therapy by promoting apoptosis and suppressing proliferation, H1373-derived tumours only responded with decreased proliferation [140]. Several groups have investigated the molecular mechanisms underlying the crosstalk between EGFR and c-Met, and it has become apparent that several partner proteins can affect EGFR-mediated phosphorylation of c-Met, including Src, MAPK and β1 integrins [84,94,100,101]. Conversely, HGF stimulation could also induce EGFR phosphorylation [138,141], pointing out the reciprocal regulation of both receptors. Other molecules that have been implicated in the crosstalk include miR-27A, sprouty, the Wnt and the mTor signaling pathways, although the precise mechanisms underlying their role in the crosstalk are unknown [142,143]. EGFR was also found to promote c-Met oncogenic function via an alternative mechanism through promoting c-Met ecto-domain shedding, leading to enhanced wound healing [95,96], a process that has previously been described to promote the oncogenic function of c-Met [144].

Similar reciprocal relationships were found between c-Met and the EGFR family members, Her2 and Her3 [91,97,102,103,104,105], IGFR [106] and RET [97]. Interestingly, Tanizaki *et al.* revealed differential functional consequences in a cell line that overexpresses c-Met upon heterodimerization between c-Met and RET, EGFR, Her2 or Her3 [97]. Loss of EGFR and Her3 resulted in a decrease in cell proliferation and survival; loss of RET decreased cell migration; and loss of Her2 decreased proliferation, survival and cell migration [97]. These results suggest some specificity in the functional outcome of c-Met partnerships with other RTKs. As c-Met is often amplified in human tumours and is a target for drug strategies, a more thorough understanding of the crosstalk between c-Met and other RTKs is necessary.

### 5.6. c-Met and Others

Other membrane spanning molecules that affect c-Met function or that are affected by c-Met interaction are mucins and death receptors. Mucins are transmembrane glycoproteins, and tumour-associated Muc1 has been shown to be important in activating signal transduction pathways to promote invasion, metastasis, proliferation and chemoresistance [145]. As opposed to this pro-tumourigenic role, in pancreatic and liver cancer cells, muc1 interacting with c-Met was found to inhibit c-Met-dependent invasion and migration [111,113,114], possibly through enhanced c-Met turnover [113]. Muc20 similarly inhibited c-Met in Hek293 and CHO-K1 cells [114]. However, in breast cancer cells, Muc1 was shown to promote c-Met-driven migration and scattering through regulation of *c-Met* mRNA expression levels [112], suggesting that tissue conditional aspects determine the consequences of the relationship between Muc1 and c-Met.

Activation of RAF/MEK/ERK and the PI3K/PDK/Akt promotes proliferation and survival, while preventing apoptosis [146]. Via activation of these signalling routes, c-Met can inhibit apoptosis, but c-Met has also been shown to influence apoptosis through interaction with cell death receptors on the cell membrane. Through interacting with Fas, c-Met prevents Fas trimerization and recruitment of an active DISC complex and, therefore, acts as an antagonist for the Fas ligand (FasL) [107,108,109]. Activation of the c-Met receptor following HGF abrogated the c-Met/Fas interaction and promoted FasL or doxorubicin-induced apoptosis [107,147]. Fatty acids or anoikis also lead to c-Met dissociation from Fas and an increase in the sensitivity to apoptosis, suggesting that the external environment, possibly through the involvement of integrins, could modulate c-Met’s function on Fas [108,109]. Similarly to Fas, DR5 was also found to be inhibited upon c-Met interaction, preventing it from interacting with TRAIL and DISC association [110]. These reports are indicative of a direct role for the inactive c-Met receptor in preventing apoptosis.

## 6. Conclusions and Future Perspectives

It is clear that c-Met function is dynamically regulated and modulated by its localization, through protein modifications and interaction with a large variety of signalling molecules, co-receptors and other membrane molecules. These interactions can greatly affect the way c-Met reacts to its ligand, HGF, but can also change the way in which c-Met will react to drug strategies that are designed to inhibit c-Met function. Inhibiting c-Met alone might not be enough to fully inhibit the c-Met signalling cascade, as other RTKs might take over this function or RTKs or other cell membrane receptors simply transactivate c-Met in a ligand-independent manner. Inhibiting multiple cell surface molecules (e.g., multiple RTKs), including c-Met, might therefore have some therapeutic potential. Trials in which c-Met and the EGFR are targeted simultaneously reveal that some NSCLC (non-small cell lung cancer) patients responded with an increased progression-free survival and overall survival, although in some patients, dependent on the molecular alterations in their genome, worse outcomes were noted [148,149]. Other combinations of RTK inhibitors for NSCLC are also being explored (summarized in [150]), as well as targeting downstream convergent signaling routes of RTKs [151]. Given that c-Met interacts with other cell surface molecules and not just other RTKs, it might also be worthwhile to investigate combination therapies in which other cell surface molecules, e.g., integrins (for which some approved drugs exist [152]), are co-targeted together with c-Met. This is also interesting in the context of RTK crosstalk, as the interaction between c-Met and integrin β1 was demonstrated to mediate EGFR inhibitor resistance [84]. Alternatively, it might be worthwhile to target common features of cell surface molecules, such as recycling or endocytosis. We have previously identified that recycling of EGFR, c-Met and integrin α5β1 was enhanced in the oncogenic setting of p53 mutations by RCP [51], leading to enhanced signalling towards Akt and ERK1/2 [51,53]. Although a better mechanistic understanding of the mutant p53 functions on RCP is required, it will be interesting to explore RCP inhibition as a means to inhibit recycling of various receptors, including c-Met, integrins and EGFR, simultaneously.

In conclusion, a better understanding of the c-Met interactors and the consequences of these interactions on c-Met signaling will help in the design of novel therapeutic strategies and in understanding the shortcomings of current strategies, including drug resistance.
